# Continuous peptide-based versus sequential standard polymeric enteral nutrition formula in critically ill patients with acute gastrointestinal injury: study protocol for a single-center, parallel-group randomized controlled trial

**DOI:** 10.3389/fmed.2026.1761334

**Published:** 2026-02-10

**Authors:** Yanhua Li, Youquan Wang, Ying Chen, Yuhan Zhang, Xinyu Li, Dong Zhang

**Affiliations:** Department of Critical Care Medicine, The First Hospital of Jilin University, Changchun, China

**Keywords:** acute gastrointestinal injury, enteral nutrition, peptide-based formula, protocol, randomized controlled trial, standardpolymeric formula

## Abstract

**Background:**

In critically ill patients with acute gastrointestinal injury (AGI), early administration of a peptide-based enteral formula (PBF) may be beneficial during the acute phase. Nevertheless, after gastrointestinal function improves, it remains unclear whether continuing a PBF or switching to a standard polymeric formula (SPF) is preferable.

**Objectives:**

To compare the efficacy and safety of continuing a PBF versus switching to an SPF from day 4 onward in critically ill patients with AGI whose gastrointestinal function has recovered or improved to AGI grade I by day 4.

**Design:**

Single-center, parallel-group, randomized, controlled, trial with 1:1 allocation, intention-to-treat analysis.

**Setting:**

Three clinical units of the Department of Critical Care Medicine at the First Hospital of Jilin University (Changchun, China).

**Participants:**

We will ultimately include 496 adult critically ill patients with AGI grade I–II who meet the eligibility criteria in the final analysis.

**Interventions:**

Continuous Peptide-Based Enteral Formula Group (PBF group): patients will receive continuous infusion of a PBF, initiated within 7 days of ICU admission. Sequential Standard Polymeric Enteral Formula Group (SPF group): patients will receive a PBF during the first 3 days in the ICU and will be transitioned to an SPF if the AGI grade is 0–I on ICU day 4.

**Primary outcome measure:**

The primary outcome is the mean daily energy intake on day 7 (kcal/kg/day). An intention-to-treat analysis will be used to assess this outcome to enhance the robustness of the results. Secondary outcomes and relevant process measures will be evaluated in accordance with the study protocol.

**Clinical trial registration:**

https://www.chictr.org.cn/showproj.html?pid=139513, Identifier ChiCTR2200056858.

## 1 Introduction

The successful implementation of early enteral nutrition (EN) can exert crucial physiological effects in critically ill patients by down-regulating systemic immune responses, reducing oxidative stress, and maintaining the intestinal microenvironment, thereby supporting cellular and organ function recovery, promoting wound healing, and ultimately improving outcomes (1–4). However, up to 60% of critically ill patients reportedly develop impairments in gastrointestinal digestion, absorption, motility, and barrier function, collectively termed acute gastrointestinal injury (AGI) (5, 6), leading to reduced or interrupted delivery of EN, subsequent malnutrition, and worsened prognosis (7–9). Therefore, selecting an appropriate EN strategy is particularly important in patients with AGI.

Choice of formula is a key component of the EN strategy for AGI. Peptide-based (short-peptide, oligomeric) enteral nutrition formulas (PBFs) are pre-digested and can be absorbed more rapidly in the intestine (10), whereas standard polymeric enteral nutrition formulas (SPFs) more closely approximate physiological conditions, stimulate digestive enzyme secretion, are absorbed more slowly, and may promote postprandial protein accretion (11). The Society of Critical Care Medicine/American Society for Parenteral and Enteral Nutrition (SCCM/ASPEN) guidelines recommend (12) initiating EN with an SPF in the Intensive Care Unit (ICU) and considering a PBF in patients with persistent diarrhea, suspected malabsorption, ischemia, or intolerance to a fiber-free formula. Our previous work (13) indicated that, in septic patients with AGI grade I–II, early use of a PBF was more suitable, whereas an SPF could be considered when EN initiation was delayed. However, in critically ill patients with gastrointestinal dysfunction, the optimal choice between continuing a PBF and transitioning to an SPF after gastrointestinal recovery (AGI 0–I) remains unclear. Current guidelines and available clinical studies offer no direct evidence to address this issue.

This study is designed to address this uncertainty by comparing the safety and efficacy of the two enteral nutrition strategies. The findings are expected to inform evidence-based selection of enteral nutrition formulas in patients with AGI.

## 2 Methods and analysis

### 2.1 Study design

**Figure 1:**
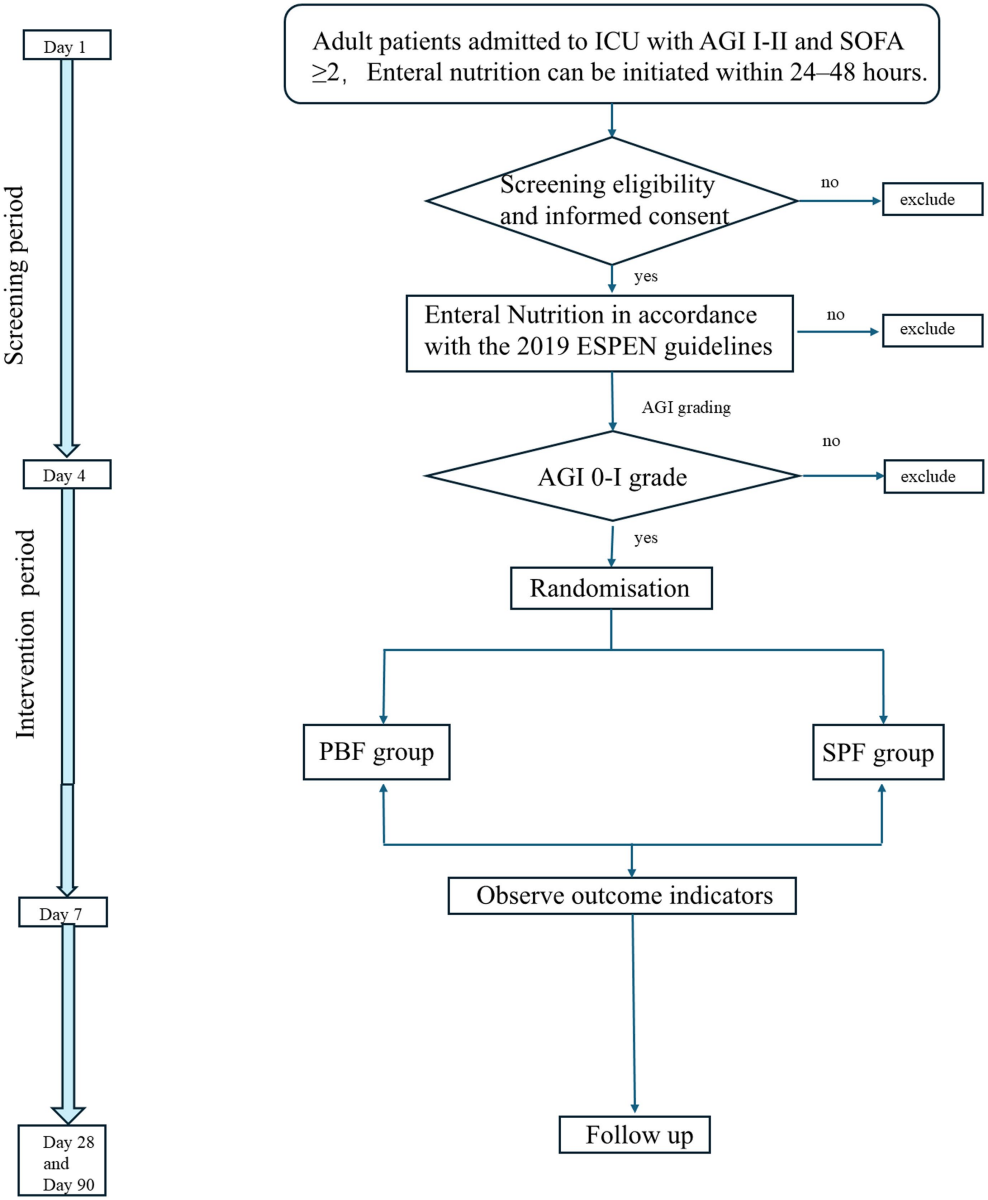
Flow diagram of patient enrollment, randomized and follow-up. ICU, Intensive Care Unit; SOFA, sequential organ failure assessment; AGI, acute gastrointestinal injury; ESPEN, European Society for Clinical Nutrition and Metabolism; PBF group, Continuous Peptide-Based Enteral Formula Group; SPF group, Sequential Standard Polymeric Enteral Formula Group.

This is an investigator-initiated, single-center (Department of Critical Care Medicine at the First Hospital of Jilin University), parallel-group, randomized controlled clinical trial. The trial has been registered with the Chinese Clinical Trial Registry (ChiCTR2200056858). The trial protocol was developed in accordance with the Standard Protocol Items: Recommendations for Interventional Trials (SPIRIT) statement (14). The flow diagram of patient enrollment, randomized and follow-up is shown in Figure 1. The final version of the protocol (version 1.0) was finalized on February 28, 2022.

### 2.2 Trial committees

The trial management committee comprises representatives from the Clinical Research Department, the Department of Critical Care Medicine, and the Biobank of the First Hospital of Jilin University. A dedicated research team within the Department of Critical Care Medicine, independent of the treating clinical team and not involved in patient care, will be responsible for outcome assessment and for clinical data collection and management. The Clinical Research Department provides methodological support, including input into study design, trial oversight, and collaboration on data management and statistical analysis, while the Biobank oversees the processing, and secure storage of biological specimens.

### 2.3 Study population

Adult critically ill patients admitted to the Department of Critical Care Medicine at the First Hospital of Jilin University who are eligible to initiate early enteral nutrition (within 24–48 h) and have AGI grade I–II with organ dysfunction will be screened for eligibility. After obtaining informed consent, feeding will follow the guideline-based protocol (15). On day 4, gastrointestinal function will be reassessed, and patients whose AGI has improved to grade 0–I will be randomized.

#### 2.3.1 Eligibility criteria

The inclusion and exclusion criteria are as follows:

##### Inclusion criteria

(1) Age ≥18 years.
(2) ICU admission with AGI grade I (gastrointestinal dysfunction with risk of failure) or grade II (gastrointestinal dysfunction) according to the 2012 European Society of Intensive Care Medicine (ESICM) (6) diagnostic criteria.
(3) Able to initiate enteral nutrition within 24–48 h of ICU admission.

##### Exclusion criteria

(1) Contraindications to enteral feeding or profound hemodynamic instability precluding any form of nutritional therapy.
(2) Concurrent oral intake.
(3) History of chronic liver disease or severe hepatic dysfunction [Child-Pugh C (16)] (Supplementary Table S1).
(4) Serum urea nitrogen increasing by >4 mmol/L per day, or renal insufficiency requiring renal replacement therapy (e.g., hemodialysis or continuous renal replacement therapy).

### 2.4 Randomization and blinding methods

Because all enrolled patients initially present with AGI grade I–II, a PBF will be administered for the first 3 study days. On day 4 of the study, gastrointestinal function will be reassessed. Patients reassessed as AGI grade 0–I will be eligible for randomization. Eligible patients will be randomized using block randomization, with a 1:1 allocation ratio to either continued a PBF (PBF group) or transition to an SPF (SPF group). The randomization sequence will be generated in R software (version 4.4.2) using permuted blocks of variable size (4 and 6).

Owing to the nature of the intervention, this is an open-label protocol, and the treating clinical team must be aware of group assignment for intervention delivery (participants may or may not be aware depending on clinical status). To minimize detection bias, feeding intolerance and other outcomes will be assessed and recorded by dedicated research staff who are independent from the ICU clinical team and do not participate in treatment decisions or nursing care.

### 2.5 Sample size

The sample size was estimated in PASS v23 (NCSS, Kaysville, UT; https://www.ncss.com/software/pass/) for the primary endpoint of energy intake on day 7 (kcal/kg/day), comparing two independent groups with equal allocation (1:1). Based on retrospective data, the SPF group had a mean ± standard deviation (SD) intake of 22.4 ± 9.825 kcal/kg/day and the PBF group had 24.9 ± 9.1 kcal/kg/day. Using a two-sided *α* = 0.05 and power (1 − *β*) = 0.90, the required sample size is *n* = 225 per group (total *N* = 450). Allowing for a 10% attrition rate, the target sample increases to *N* = 495. To maintain balance between the two treatment groups under block randomization with variable block sizes of 4 and 6, the final planned sample size is *N* = 496.

### 2.6 Study interventions

Following enrollment, all patients will receive a PBF during the run-in phase (study days 1–3). On study day 4, gastrointestinal function will be reassessed. Participants meeting AGI grade 0–I criteria are randomized (1:1) to one of two intervention arms: continuous Peptide-Based Enteral Formula Group (PBF group) and Sequential Standard Polymeric Enteral Formula Group (SPF group). All other aspects of ICU care follow contemporary best practice and local guidelines. Table 1 shows the schedule of enrolment, interventions, and assessments based on the SPIRIT 2013 statement (14). Detailed reporting checklists are provided in the Supplementary Table S2 (SPIRIT 2013 checklist).

**Table 1:** Schedule of enrolment, interventions, and assessments.

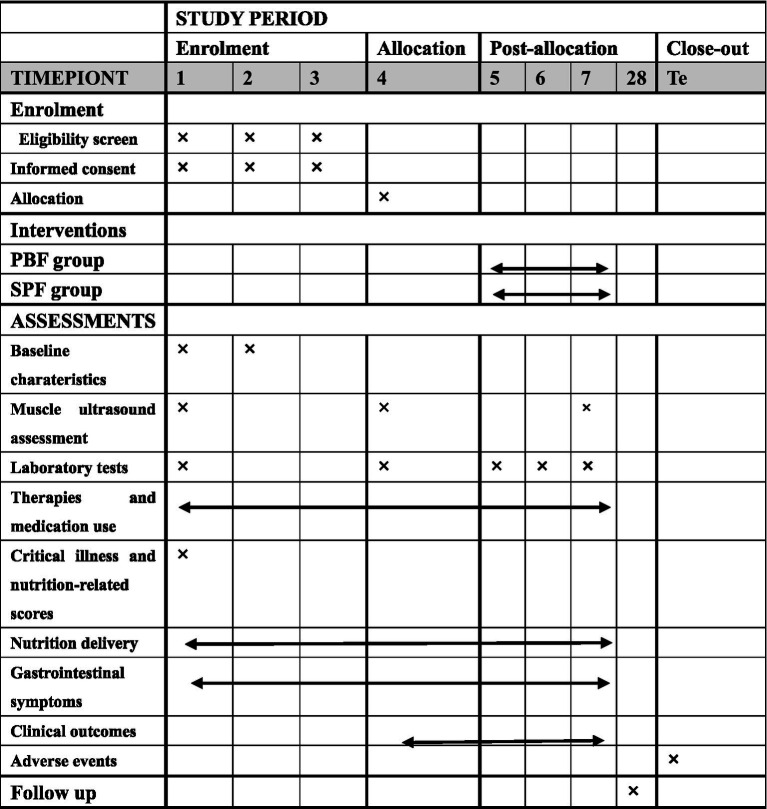

PBF group, Continuous Peptide-Based Enteral Formula Group; SPF group, Sequential Standard Polymeric Enteral Formula Group.

#### 2.6.1 Arm#1: continuous Peptide-Based Enteral Formula Group (PBF group)

Intervention. Continue the PBF initiated during the run-in phase.

Administration mode. Continuous pump infusion via gastric (preferred) or post-pyloric feeding tube.

#### 2.6.2 Arm#2: Sequential Standard Polymeric Enteral Formula Group (SPF group)

Intervention. Transition from a PBF to an SPF after randomization.

Administration mode. Continuous pump infusion (gastric or post-pyloric).

#### 2.6.3 General management

All other aspects of patient management will follow standard clinical practice. This includes, but is not limited to, monitoring of vital signs, feeding tolerance, laboratory testing, fluid resuscitation, mechanical ventilation, and vasopressor support, as deemed necessary by the attending clinical team. No study-directed interventions will be applied to any domain of care other than nutritional support. All concomitant treatments will be determined by the responsible clinicians and documented in the medical record.

Nutrition protocol. Enteral nutrition will be delivered in accordance with the 2019 European Society for Clinical Nutrition and Metabolism (ESPEN) guidelines (15). Because indirect calorimetry is not used in this study, the energy target will be set using a simplistic weight-based equation of 25–30 kcal/kg/day consistent with the 2016 ASPEN guideline (12) and several large clinical studies (17, 18), The protein target is 1.3 g/kg/day (15). For patients with obesity Body Mass Index (BMI) ≥ 30 kg/m^2^, adjusted body weight (AdjBW) will be used for energy and protein calculations, as recommended by 2019ESPEN guidelines (15). AdjBW = Ideal Body Weight (IBW) + 0.33 × (Actual Body Weight − IBW), where IBW is estimated using a BMI-based method [IBW = 2.2 × BMI + 3.5 × BMI × (height – 1.5 m)].

Food intolerance (FI) will be defined in accordance with the terminology and definition framework proposed by the ESICM (6) Working Group on Abdominal Problems in 2012. FI management in both groups will be conducted in accordance with the recommendations of the 2022 Evidence-Driven Feeding Guidelines for Nutrition in Critically Ill Patients (NEED) (17).

Parenteral nutrition (PN). In patients who do not tolerate full-dose enteral nutrition during the first week in the ICU, the safety and potential benefits of PN will be assessed case-by-case, with the final decision made by the attending physician.

#### 2.6.4 Ultrasound training for both groups

All enrolled patients will undergo ultrasound examinations on study days 1, 4, and 7. Measurements will be performed according to a standardized protocol and recorded in the case report form. Prior to enrolling the first patient, dedicated researchers who must have completed China Critical Care Ultrasound Study Group (CCUSG) training and obtained a certificate will receive ultrasound training. Ultrasound training will be conducted by certified trainers from the CCUSG. Standardized measurement protocols and instructional videos are available through the platforms https://studio.youtube.com/playlist/PL-INecaASaeDazZVy8ivoiLw0ycG0cT0_/videos.

### 2.7 Study outcomes and their measures

#### 2.7.1 Primary outcome definition and measures

Energy intake on Day 7, defined as the actual caloric intake on study Day 7 (kcal/kg/day). For patients with BMI > 30 kg/m^2^, an adjusted body weight will be used.

#### 2.7.2 Secondary outcome definition and measures

1. Protein intake on Day 7, defined as the actual protein delivered on study Day 7 (g/kg/day). For patients with BMI > 30 kg/m^2^, an AdjBW will be used.
2. Occurrence of FI between ICU days 4–7, FI is defined as (6) present if any of the following criteria are met: ① Vomiting: any visible regurgitation of gastric contents, irrespective of volume. ② High gastric residual volume (GRV): any single GRV > 200 mL. ③ Diarrhea: ≥3 loose or liquid stools per day and stool weight >200–250 g/day or volume >250 mL/day. ④ Gastrointestinal (GI) bleeding: any intraluminal GI bleeding confirmed by gross blood in vomitus, gastric aspirate, or stool. ⑤ Insufficient enteral energy delivery: failure to achieve ≥20 kcal/kg body weight/day via the enteral route within 72 h after initiation/attempt of enteral feeding. ⑥ Cessation of enteral nutrition: enteral feeding discontinued for any clinical reason. Outcome recording. The presence or absence of FI will be assessed once per day on ICU days 4–7. In addition to the composite FI outcome, individual FI components will be reported and analyzed separately.
3. Change in nutritional biomarkers. Defined as serum albumin and prealbumin concentrations measured on study day 1 (baseline) and day 7. Reporting values (albumin in g/L; prealbumin in mg/L) at each time point; change calculated as Day 7 − Day 1.
4. Skeletal muscle status. Muscle mass change will be assessed using the following sonographic metrics on ICU days 1, 4, and 7 after admission: upper-limb muscle thickness; quadriceps muscle thickness; rectus femoris cross-sectional area (RF-CSA).
5. Intestinal injury biomarkers. Between-group differences in intestinal injury markers will be evaluated using serum/plasma concentrations of D-lactate, intestinal fatty-acid binding protein (I-FABP), and diamine oxidase (DAO) on study Day 1 and Day 7. *Measurement & reporting:* assays performed per manufacturer instructions; values reported in assay-specified units. Changes from baseline (Day 7–Day 1) and between-group differences in change will be analyzed.
6. New infections in the ICU: Defined as infections newly acquired ≥48 h after ICU admission.
7. ICU length of stay.
8. Ventilator-free days: Ventilator-free days within 28 days in the ICU.
9. ICU hospitalization cost. Defined as the total direct medical cost accrued during the index ICU stay, from ICU admission (time 0) to ICU discharge. Extracted from the hospital billing system/financial data warehouse using patient identifiers and ICU encounter numbers.
10. 28-day mortality: Assessed on day 28 after ICU admission.
11. 90-day mortality: Assessed on day 90 after ICU admission.

#### 2.7.3 Duration of treatment and follow-up

The study intervention will continue for 28 days following ICU admission, unless the patient is discharged from the ICU, discharged from the hospital, or dies earlier. All randomized patients will be followed for 90 days after ICU admission, unless death occurs first. If a patient remains hospitalized on day 90, follow-up will be conducted and outcomes will be recorded based on the patient’s status on day 90.

#### 2.7.4 Reasons for withdrawal/discontinuation

If any serious adverse events related to the study occur during the trial, early withdrawal from the study will be permitted. In addition, if a patient or their legal representative requests withdrawal from the trial for any reason, early withdrawal will be allowed without affecting the patient’s future medical care from the investigators or study site. Efforts will be made to follow up all randomized patients to the extent permitted by their consent.

#### 2.7.5 Exposure metrics, contamination and intervention fidelity

##### 2.7.5.1 Exposure metrics

All participants receive a short-peptide formula from enrollment through study Day 3; this run-in period is not included in exposure analyses and is used solely for baseline standardization and tolerance assessment. Randomization occurs on Day 4 (D4). The exposure assessment window is D4–D7. We will record:

- Duration of the exposure window (hours): elapsed time from randomization (D4) to 23:59 on D7 or to ICU transfer/discharge or death, whichever occurs first.
- Concordant feeding hours: cumulative hours during which the randomized formulation was actually administered.
- Adherence (%) = (concordant feeding hours ÷ exposure window hours) × 100. Interruptions (e.g., procedures, intolerance) will be time-stamped; calculations use actual accrued hours within the window.

##### 2.7.5.2 Contamination

Within the exposure window, any administration (any dose, any single occasion) of a formulation not matching the randomized assignment is classified as contamination (patient-level binary; event-level counts recorded). To minimize contamination, the study coordinator will perform daily reconciliation of administered formula against the randomization list and notify the bedside team immediately upon discrepancy.

##### 2.7.5.3 Intervention fidelity (adherence)

Fidelity is defined as complete execution of the feeding plan with the randomized formulation throughout the exposure window. Primary adherence is reported as the proportion of participants with 100% concordance; a secondary, continuous measure uses the adherence % defined above. If the attending physician changes the formulation for clinical reasons (e.g., fluid restriction, significant feeding intolerance), the reason must be documented, along with: start/stop timestamps of non-assigned formula, total time on non-assigned formula, and total exposure-window hours. Data sources: electronic medical records and clinical study report forms.

##### 2.7.5.4 CONSORT reporting

We will report (i) contamination rate, (ii) intervention fidelity, and (iii) duration of the exposure window (hours).

### 2.8 Data collection and analysis

#### 2.8.1 Data collection

We will use electronic forms to collect relevant clinical information of enrolled patients. Once the data is entered, it cannot be modified. The data entry will be completed by full-time scientific research team members of the Department of Critical Care Medicine of the First Hospital of Jilin University. They will not participate in the clinical treatment of patients and the analysis of the final data.

#### 2.8.2 Data analysis

All statistical analyses will be conducted in accordance with a pre-specified statistical analysis plan. The primary analysis will follow the intention-to-treat (ITT) principle The Full Analysis Set (FAS) will include all participants who were successfully randomized and entered the intervention period, provided that they have a valid assessment of the primary outcome (caloric intake on day 7) or sufficient information to derive this value. The Per Protocol Set (PPS) will be a subset of the FAS, including participants who received the allocated intervention for at least 72 h and had no major protocol deviations. The PPS will be used for sensitivity analyses to assess the robustness of the primary findings. The SS will include all participants who received at least one dose of the study intervention and will be used for safety analyses.

The primary outcome, caloric intake on day 7 (continuous variable), will be analyzed using a linear regression model. The model will include the intervention group and the predefined covariates (APACHE II score, BMI, AGI grade, and primary diagnosis) as fixed effects. As this is a single-center randomized controlled trial, no random effects will be specified. To account for potential model misspecification and heteroscedasticity, heteroskedasticity-robust (sandwich) standard errors will be used for statistical inference. If the model residuals show substantial deviation from normality or are influenced by outliers, data transformation (for example, logarithmic transformation) or a nonparametric bootstrap method will be applied in sensitivity analyses to assess the robustness of the results.

Continuous secondary outcomes (e.g., the number of days without a ventilator in the ICU) will be analyzed analogously using linear regression models; secondary binary outcomes (e.g., FI) will be analyzed using a generalized linear model (GLM) with a logit link function. The model will include the intervention group and the pre-specified covariates as fixed effects. As this is a single-center study without inter-center or clustering structures, no random effects will be specified. In addition, because FI is a composite secondary endpoint, each FI component will be reported and analyzed separately alongside the composite FI outcome.

Continuous variables will be summarized as mean ± SD if normally distributed, or as median and interquartile range (IQR, P25–P75) if not normally distributed. Between-group differences in continuous variables will be tested using the independent samples t-test for normally distributed data or the Mann–Whitney U test for non-normally distributed data. Categorical variables will be compared using the Chi-square test.

To assess whether between-group differences in caloric adequacy and tolerance reflect intrinsic physiological effects of the enteral formulas or are confounded by differences in feeding tolerance management, we will compare key nutrition-support process indicators (time to EN initiation; FIS-guided feeding-rate adjustments every 4–6 h; use of prokinetic and/or anti-diarrheal agents; post-pyloric feeding) between groups during the intervention period and will add these as covariates for adjusting confounding factors. At the same time, we will conduct sensitivity analyses to test the robustness of the results.

Sensitivity analyses will include: (I) an unadjusted GLM including only the treatment factor; (II) a covariate-augmented GLM including the prespecified covariates APACHE II score, AGI grade, BMI, primary diagnosis as well as baseline mechanical ventilation. (III) Key treatment indicators include time to initiation of enteral nutrition, feeding-rate adjustments at 4–6-h intervals based on the FIS score, use of prokinetics or antidiarrheals, and use of post-pyloric feeding. All covariates are prespecified and will be retained in the models regardless of statistical significance.

Adjusted odds ratios (ORs) with 95% confidence intervals (CIs) will be reported for binary outcomes, with standardized marginal risk differences provided as supplementary estimates. The primary endpoint will be tested at a two-sided *α* = 0.05 level without multiplicity adjustment. For prespecified secondary clinical endpoints, the false discovery rate (FDR) will be controlled at q = 0.05 using the Benjamini–Hochberg procedure based on two-sided *p* values from the specified models.

Process outcomes (such as fidelity, exposure, and contamination) will be treated as exploratory outcomes and summarized descriptively with 95% CIs; no hypothesis testing or multiplicity correction will be applied to these exploratory analyses.

All analyses were performed using R software (version 4.4.2; R Foundation for Statistical Computing, Vienna, Austria) within RStudio (version 2024.12.1+563; Posit Software, Boston, MA, USA). Two-sided *p* values <0.05 were considered statistically significant.

Given the potential for confounding and co-interventions inherent in nutritional interventions, strong causal inferences will not be made. Primary and secondary analyses are prespecified to be interpreted as estimates of association following adjustment for predefined covariates and sensitivity analyses. Any residual between-group differences will be reported cautiously.

The robustness of between-group differences will be evaluated after adjustment for prespecified clinical covariates and key management-related variables (e.g., tolerance management and feeding strategies). Attenuation of associations after adjustment may suggest partial explanation by management factors, whereas consistency of findings across sensitivity analyses may be considered compatible with potential formula-related differences; however, all conclusions will remain cautious.

#### 2.8.3 Missing data and interpolation strategies

The proportion of missing data for the primary outcome (caloric intake on day 7) is expected to be approximately 15%, mainly due to discharge, transfer, or incomplete records. The primary analysis will be conducted in the FAS using multiple imputation (MI) under the missing-at-random (MAR) assumption. The imputation model will include treatment group, APACHE II score, BMI, AGI grade, primary diagnosis, and other variables related to missingness or the outcome.

As sensitivity analyses, a complete-case analysis (including only participants with observed day-7 outcomes) will be performed. In addition, PPS analysis and delta-adjusted MI analyses will be conducted to assess the robustness of the results under alternative missing data assumptions.

If the results are consistent across analyses, the study findings will be considered robust.

#### 2.8.4 Confidentiality and data security

All collected data will be stored in secure electronic spreadsheets. No individual other than the data administrator will have the authority to modify or export patient data or information. Strict measures will be taken to ensure the confidentiality and security of all patient information. The final trial dataset will be accessible only to the principal investigator, the study statisticians, and authorised members of the trial management committee at the First Hospital of Jilin University. No contractual agreements with sponsors or third parties limit the access of the investigators to the final dataset or their ability to analyse and publish the results.

### 2.9 Adverse events

Adverse events are defined according to the CTCAE by the U. S. National Cancer Institute as any unfavorable medical occurrence in a patient receiving the study intervention, which does not necessarily have a causal relationship with the treatment.

Adverse events that may occur during the use of EN in this study include nausea, vomiting, reflux, and gastrointestinal bleeding. Patients will be closely monitored, and appropriate treatment will be provided if symptoms occur. Measures such as elevating the head of the bed will be taken to reduce the risk of aspiration.

## 3 Discussion

In patients with impaired intestinal function, the choice of enteral formula is a key determinant of successful EN delivery and of realizing its therapeutic advantages. Multiple guidelines recommend (3, 4, 12, 15) that an SPF should be used as first-line for most critically ill adults, while a PBF may be considered in patients with persistent diarrhea, suspected malabsorption, ischemia, or inadequate response to fiber. However, for patients with intestinal dysfunction after partial recovery of gut function, it remains unclear whether and when to transition from a PBF to an SPF; current guidelines provide no specific recommendations on timing or criteria for such switching. This study protocol is designed to address this evidence gap.

Experimental studies in rats (19–21) indicate that exposure of the colon to N-formyl-methionyl-leucyl-phenylalanine (fMLP) increases the expression of oligopeptide transporters, e.g., Peptide Transporter (PEPT) family, which may aggravate colonic mucosal injury. PBFs could confer a gastrointestinal protective effect by competitively inhibiting fMLP transport and/or leveraging higher transport efficiency through the same carriers, thereby reducing epithelial damage. Additional evidence suggests that (10), compared with SPFs, PBFs accelerate intestinal digestion and absorption of protein, enhance postprandial amino-acid availability, and may increase the rate of skeletal-muscle protein synthesis. Our prior work further showed that (8, 22), versus SPFs, PBFs enabled faster achievement of nutritional targets via EN and were associated with a lower incidence of feeding intolerance. Recent evidence from our team (13) in patients with AGI grade I–II who initiated EN within 48 h, PBFs were associated with greater caloric adequacy and lower incidences of gastric residuals and diarrhea; conversely, among those who initiated EN between days 3–7, SPFs achieved higher caloric adequacy, with no significant differences in gastric residuals or diarrhea between group. These data imply a time-dependent formula preference—PBFs may be more appropriate early, whereas SPFs may be preferable on days 3–7. However, because the study was retrospective, selection bias related to baseline gastrointestinal function cannot be excluded. Accordingly, we designed a prospective, randomized, controlled study to further evaluate, among AGI grade I–II patients who receive PBFs feeding for the first 3 days and whose intestinal function recovers to or remains at AGI 0–I, whether continuing PBFs versus switching to SPFs from day 4 differs in caloric and protein intake, nutritional biomarkers, and intestinal injury markers. We also planned to assess trajectories of muscle mass to clarify whether peptide formulas better support muscle protein synthesis and mitigate muscle loss in critical illness. Key clinical outcomes include mortality, ICU length of stay, ventilator-free time, and the incidence of ICU-acquired infections. Overall, this study will compare the two formulas across nutritional adequacy, feeding tolerance, intestinal protection, muscle preservation, and clinical outcomes, providing clinically meaningful evidence.

This study will exclude patients with chronic liver diseases and severe liver and kidney dysfunction, as these conditions significantly alter energy/protein metabolism, fluid-electrolyte management, and gastrointestinal function. This exclusion is intended to enhance patient safety and reduce metabolic and clinical heterogeneity that could interfere with nutritional delivery and feeding tolerance outcomes. Therefore, our findings are most applicable to ICU patients without chronic liver diseases or severe liver and kidney dysfunction. To improve transparency regarding generalizability, we will report the number of patients excluded due to chronic liver disease or severe renal/hepatic dysfunction in the final report.

This study will use a 3-day run-in with a peptide-based formula and will be randomized only patients who AGI grade 0–I by day 4. This approach standardizes early enteral nutrition and avoids exposing patients with compromised GI function to potentially unsuitable intact-protein formula; however, it may introduce selection bias and reduce external validity. Therefore, our results primarily apply to AGI I–II patients who can tolerate peptide-based enteral nutrition during the early phase and show rapid improvement of GI function, and may not be generalizable to patients with persistent or worsening AGI II.

This study has several notable strengths. 1. To our knowledge, this is the first large randomized controlled trial to address whether, in critically ill patients with AGI whose intestinal function has recovered or at least not worsened, a transition from a PBF to an SPF is necessary. 2. We selected actual caloric intake as the primary endpoint to directly capture the impact of formula type on achieved nutrition, and we complemented this with a broad set of secondary outcomes—including actual protein intake, feeding tolerance, nutritional serum biomarkers, intestinal injury markers, trajectories of muscle mass, and key clinical endpoints—to provide a comprehensive and systematic evaluation. 3. Conducting the trial at a single center is expected to enhance consistency in the feeding protocol and ultrasound measurements. 4. To reduce heterogeneity and confounding, we excluded patients with hepatic or renal disease and those receiving extracorporeal therapies, conditions known to influence metabolism and nutritional requirements (23). 5. Moreover, clinical data are collected by an independent research team that is not involved in patient care, randomization, or data analysis, thereby minimizing subjective bias.

This study also has important limitations. 1. The single-center design may limit external validity and generalizability. 2. Our primary endpoint—caloric intake on ICU Day 7—introduces a risk of missing data among patients who are discharged or die before Day 7; these cases require imputation, which may deviate from true values. 3. Despite standardized training based on the guideline, some team-level subjectivity in implementation is unavoidable and could affect delivered feeding volumes or tolerance management. Although we will adjust for prespecified covariates, co-interventions and variability in feeding strategies may still influence the results, and residual confounding cannot be excluded. Our findings will be interpreted cautiously. 4. Finally, due to resource constraints, indirect calorimetry was not used to set energy targets, which may reduce the precision of prescribed goals.

## 4 Ethics and dissemination

### 4.1 Confidentiality issues

Processes and safeguards will be in place to protect all information stored in the web-based system. Data access will be secure and restricted to authorized users. Network protections will prevent unauthorized access or transfer of data. Data security is ensured through multiple measures: servers are protected by firewalls, physical access is restricted, and the server is used exclusively for database management. All patient data will be deidentified to protect privacy, and no personal identifiers will appear in any publication. Confidentiality will be maintained in accordance with applicable regulations.

### 4.2 Potential risks and benefits

In this study, enteral feeding was administered in accordance with the most recent international guidelines. Feeding intolerance was monitored in real time and managed promptly, with the aim of reducing intolerance-related adverse outcomes.

### 4.3 Dissemination policy

The study results will be disseminated through peer-reviewed journals and scientific conferences. Neither patients nor the public were involved in the design, conduct, reporting, or dissemination of this research. Not commissioned; externally peer reviewed.
